# Stage-dependent alterations of Sonoclot coagulation profiles across the spectrum of diabetic peripheral neuropathy

**DOI:** 10.3389/fendo.2026.1876875

**Published:** 2026-07-13

**Authors:** Yunqing Zhu, Ziyang Shen, Rujin Zang

**Affiliations:** 1 Department of Endocrinology, Nanjing First Hospital, Nanjing Medical University, Nanjing, China; 2 Department of Pediatrics, Affiliated Taizhou People’s Hospital of Nanjing Medical University, Taizhou, China

**Keywords:** clot rate, diabetic peripheral neuropathy, Sonoclot, subclinical diabetic peripheral neuropathy, type 2 diabetes mellitus

## Abstract

**Background:**

Microvascular hypercoagulability is a primary driver of diabetic peripheral neuropathy (DPN). This study aimed to evaluate comprehensive whole-blood viscoelastic profiles using the Sonoclot analyzer across distinct clinical stages of DPN.

**Methods:**

This cross-sectional study consecutively enrolled 289 hospitalized patients with type 2 diabetes mellitus (T2DM). Based on the Toronto Clinical Neuropathy Score (TCNS) and nerve conduction studies, participants were classified into non-DPN, subclinical DPN (sDPN), and confirmed DPN groups. Sonoclot-derived parameters were quantitatively assessed. Multivariable logistic regression, restricted cubic spline (RCS) analysis, and receiver operating characteristic (ROC) curve analysis were performed to evaluate diagnostic performance.

**Results:**

Among the 289 participants, 93 (32.2%) were diagnosed with sDPN and 70 (24.2%) with confirmed DPN. CR levels did not differ significantly between the non-DPN and sDPN groups, whereas markedly elevated CR levels were observed in patients with confirmed DPN. After adjustment for age, diabetes duration, HbA1c, BMI, and platelet count, CR remained independently associated with DPN (odds ratio 1.154, 95% CI 1.075–1.239). RCS analysis demonstrated a significant nonlinear association between CR levels and the presence of DPN. ROC analysis identified an optimal CR cutoff value of 31.1 (AUC = 0.729), with a sensitivity of 47.1% and a specificity of 98.9%. The moderate sensitivity suggests limited utility as a standalone screening marker.

**Conclusion:**

Sonoclot-derived CR is independently associated with clinically confirmed DPN. Longitudinal studies are required to clarify the temporal nature of this association.

## Introduction

Type 2 diabetes mellitus (T2DM) represents a major global public health challenge (1). Among the chronic microvascular complications associated with T2DM, diabetic peripheral neuropathy (DPN) is the most prevalent and debilitating, affecting up to 50% of patients during their lifetime (2). Clinically, DPN is characterized by a length-dependent, distal-to-proximal degeneration of peripheral nerve fibers, resulting in neuropathic pain, sensory loss, and impaired proprioception (3). Beyond its profound impact on quality of life, DPN is a major contributor to diabetic foot ulceration and non-traumatic lower-extremity amputation and is independently associated with increased all-cause mortality (4).

Importantly, the development of DPN is preceded by a prolonged subclinical phase, referred to as subclinical DPN (sDPN), during which electrophysiological abnormalities and endoneurial microvascular dysfunction emerge in the absence of overt neurological symptoms (5). Once symptomatic DPN develops, neural injury is often irreversible and current therapeutic approaches remain largely limited to symptomatic management rather than disease modification (6). Therefore, identifying sensitive biomarkers capable of distinguishing different DPN related clinical profiles represents a critical clinical priority.

The pathogenesis of DPN is multifactorial and involves metabolic dysregulation and microvascular ischemia. Peripheral nerves depend heavily on an intact microvascular network to maintain adequate oxygen and nutrient delivery (7, 8). In T2DM, chronic hyperglycemia is associated with endothelial dysfunction and disrupted hemostatic balance, thereby promoting a chronic prothrombotic state (9). This hypercoagulable milieu has been linked to capillary thrombosis and microvascular narrowing, leading to nerve ischemia and progressive axonal injury (10). Recent studies have further demonstrated that coagulation abnormalities are independently associated with both the presence and severity of DPN (9).

Conventional coagulation assays, including prothrombin time (PT) and activated partial thromboplastin time (APTT), provide only limited information regarding the initiation phase of coagulation in platelet-poor plasma and therefore fail to capture the dynamic whole-blood properties of *in vivo* hemostasis (11). Consequently, whole-blood viscoelastic testing (VET), particularly thromboelastography (TEG), has increasingly been used to evaluate global coagulation function. Previous studies have shown that TEG-derived indices reflecting fibrinogen activity, including the k-value and angle α, are significantly associated with the development of both subclinical and confirmed DPN (12, 13).

Nevertheless, TEG remains limited by its cup-and-pin oscillation mechanism, which may be insufficiently sensitive to subtle viscoelastic alterations associated with early platelet hyperreactivity and microfibrin formation (14). The Sonoclot analyzer is an advanced point-of-care viscoelastic testing system that may address several of these limitations. By using a vertically oscillating probe within a whole-blood sample, Sonoclot continuously monitors the entire coagulation process and generates three major parameters: activated clotting time (ACT), clot rate (CR), and platelet function (PF) (15). Comparative studies suggest that Sonoclot may be more sensitive than TEG in detecting accelerated fibrin formation, abnormal fibrinogen–platelet interactions, and platelet hyperactivity in patients with metabolic disorders (14). Despite known associations between systemic hypercoagulability and DPN, the precise viscoelastic alterations that distinguish subclinical DPN from confirmed DPN remain a critical evidence gap. We hypothesized that Sonoclot-derived parameters, given their sensitivity to fibrin kinetics, may help characterize hemostatic profiles associated with different DPN status and neuropathy severity.

Accordingly, this study aimed to systematically evaluate Sonoclot coagulation profiles in patients with T2DM without neuropathy, with sDPN, and with confirmed DPN. We further sought to determine whether Sonoclot-derived parameters could serve as sensitive biomarkers for distinguishing sDPN from confirmed DPN.

## Research design and methods

### Study participants

This cross-sectional study was conducted at the Department of Endocrinology, Nanjing First Hospital, between November 2024 and May 2025. Patients with T2DM were consecutively recruited upon admission according to the 2024 American Diabetes Association diagnostic criteria. Eligible participants were required to have a confirmed diagnosis of T2DM and available Sonoclot measurements.

Exclusion criteria were as follows (1): type 1 diabetes mellitus or other specific types of diabetes (2); severe hepatic dysfunction (ALT or AST ≥3 times the upper limit of normal) or renal dysfunction (eGFR <45 mL/min/1.73 m²) (3); active malignancy (4); other causes of peripheral neuropathy, including chronic alcoholism, vitamin B12 deficiency, or exposure to neurotoxic agents (5); age >85 years, psychiatric disorders, or inability to complete study procedures; and (6) pregnancy or lactation.

A total of 289 patients with T2DM were included in the final analysis. Because this study was exploratory and based on consecutive patient recruitment, a formal *a priori* sample size calculation was not performed. A *post-hoc* power analysis indicated that the study had >80% power to detect association between CR and DPN at an alpha level of 0.05. The study protocol was approved by the Institutional Review Board of Nanjing First Hospital (KY20250811-KS-02) and conducted in accordance with the Declaration of Helsinki. Written informed consent was obtained from all participants.

### Assessment of DPN

DPN was assessed using the Toronto Clinical Neuropathy Score (TCNS) combined with nerve conduction studies (16). All participants underwent standardized electrophysiological testing. Motor nerve conduction studies were performed on the ulnar, median, and tibial nerves, while sensory conduction studies were conducted on the ulnar, median, and sural nerves on the non-dominant side. Body temperature was maintained at approximately 34 °C during testing. Abnormal nerve conduction was defined as one or more abnormal parameters in at least two tested motor or sensory nerves compared with laboratory reference values (17).

Nerve conduction assessment included conduction velocity, amplitude, and latency of both motor and sensory nerves. Participants with a TCNS score of 0 and normal nerve conduction studies were classified as non-DPN. Participants with a TCNS score of 0 but abnormal nerve conduction findings were classified as having sDPN. Confirmed DPN was defined as a TCNS score >5 combined with abnormal nerve conduction findings.

### Clinical and laboratory assessments

Baseline demographic and clinical characteristics, including age, sex, height, weight, duration of diabetes, smoking status, alcohol consumption, hypertension, coronary heart disease (CHD), and antidiabetic medication use, were obtained from medical records and participant interviews. Smoking status was categorized as never, former (cessation >1 year), or current smoking. Alcohol consumption was categorized as never, former (cessation >1 year), or current drinking within the previous 12 months. Body mass index (BMI) was calculated as weight divided by height squared (kg/m²). Blood pressure values represented the average of three measurements.

Following an overnight fast of at least 8 h, venous blood samples were collected for biochemical analyses. Glycated hemoglobin A1c (HbA1c) was measured using high-performance liquid chromatography (D-10; Bio-Rad, Hercules, CA, USA). Fasting plasma glucose (FPG), creatinine (Cr), uric acid (UA), total cholesterol (TC), triglycerides (TG), high-density lipoprotein cholesterol (HDL-C), and low-density lipoprotein cholesterol (LDL-C) were quantified using automated enzymatic colorimetric assays (Hitachi 7180; Hitachi, Tokyo, Japan). Estimated glomerular filtration rate (eGFR) was calculated using the Chronic Kidney Disease Epidemiology Collaboration (CKD-EPI) equation.

Sonoclot measurements were performed by trained laboratory technicians according to the manufacturer’s standard operating procedures. The analyzer underwent routine daily calibration and internal quality-control procedures before sample testing. Blood samples were collected after an overnight fast and analyzed within the time frame recommended by the manufacturer. Laboratory personnel performing Sonoclot measurements were blinded to participants’ clinical characteristics and neuropathy status. To reflect real-world clinical conditions, participants receiving routine antiplatelet therapies did not discontinue their medications prior to viscoelastic testing. Prior to testing, the Sonoclot coagulation analyzer was preheated according to the manufacturer’s instructions. Subsequently, 1 mL of venous whole blood was transferred into the sample cup positioned within the reaction chamber. The Sonoclot system continuously monitored the coagulation process and generated three primary parameters: ACT, CR, and PF.

### Statistical analysis

All statistical analyses were performed using R software (version 4.5.1; R Foundation for Statistical Computing, Vienna, Austria). Continuous variables are presented as mean ± standard deviation (SD) for normally distributed data or median (interquartile range) for non-normally distributed data, as assessed by the Shapiro–Wilk test. Categorical variables are expressed as frequencies and percentages.

Comparisons among the non-DPN, sDPN, and DPN groups were conducted using Welch’s ANOVA or the Kruskal–Wallis test for continuous variables and the chi-square test or Fisher’s exact test for categorical variables, as appropriate. Spearman correlation analysis was performed to evaluate the associations between CR levels and clinical or laboratory parameters.

Multivariable logistic regression models were constructed to examine the independent association between CR and DPN. Based on the directed acyclic graph framework, age, diabetic duration, HbA1c, BMI, and platelet count were identified as essential confounding variables requiring adjustment in analyses involving Sonoclot-derived parameters. Restricted cubic spline (RCS) analysis with four knots was subsequently performed within the logistic regression framework to characterize and visualize the potential nonlinear association between CR levels and DPN risk. Goodness-of-fit was evaluated using the Hosmer–Lemeshow test, which demonstrated adequate model fit (P > 0.05). Multicollinearity was assessed using variance inflation factors (VIFs), and all variables showed acceptable VIF values (VIF < 2). Receiver operating characteristic (ROC) curve analysis was conducted to evaluate the discriminatory performance of CR for DPN. A two-sided P value <0.05 was considered statistically significant.

## Results

### Characteristics of the study participants

Among the 289 enrolled participants, 70 (24.2%) were diagnosed with confirmed DPN and 93 (32.2%) with subclinical DPN. The baseline clinical and laboratory characteristics of the study population are summarized in **Table 1**.

**Table 1:** Baseline characteristics of patients with versus without diabetic peripheral neuropathy.

Variables	Patients without DPN (N = 126)	Patients with sDPN (N = 93)	Patients with DPN (N = 70)	P value
Male n,(%)	80 (63.5%)	63 (67.7%)	40 (57.1%)	0.38
Age (years)	54.41 ± 12.38	55.91 ± 13.12	69.16 ± 10.06 *#	<0.001
Diabetic duration (years)	7.00 (2.00, 12.00)	10.00 (3.00, 15.00)	10.00 (8.00, 20.00) *#	<0.001
SBP (mmHg)	132.93 ± 18.90	139.33 ± 20.03	134.57 ± 22.14	0.063
DBP (mmHg)	85.03 ± 10.87	88.26 ± 11.29	80.04 ± 10.93 *#	<0.001
BMI (kg/m2)	25.39 ± 3.47	25.69 ± 4.45	25.32 ± 3.60	0.792
WBC (10^9/L)	6.40 ± 1.76	6.83 ± 1.91	6.52 ± 1.99	0.246
HGB (g/L)	138.47 ± 14.97	143.33 ± 14.24	129.91 ± 17.10 *#	<0.001
RBC (10^9/L)	4.57 ± 0.50	4.72 ± 0.58	4.28 ± 0.57 *#	<0.001
PLT (10^9/L)	198.00 (165.00, 239.00)	189.00 (167.00, 227.00)	187.00 (156.25, 226.00)	0.491
HbA1c (%)	8.58 ± 1.46	9.08 ± 1.61	9.44 ± 1.80 *	0.001
ALT (U/L)	20.00 (15.00, 29.00)	21.00 (15.00, 31.00)	16.00 (12.00, 22.75) *#	0.002
AST (U/L)	19.00 (16.00, 23.00)	19.00 (15.00, 24.00)	18.00 (14.00, 22.00)	0.195
ALB (g/L)	40.83 ± 2.75	41.00 ± 3.18	38.23 ± 3.93 *#	<0.001
FBG (mmol/L)	7.32 ± 2.62	7.48 ± 2.24	8.29 ± 2.92 *	0.036
UA (mmol/L)	299.13 ± 84.42	303.60 ± 90.97	310.99 ± 88.26	0.662
TC (mmol/L)	4.68 ± 1.22	4.95 ± 1.51	4.28 ± 1.11#	0.005
TG (mmol/L)	1.50 (1.02, 2.19)	1.44 (1.05, 2.32)	1.23 (0.95, 1.73)	0.053
HDL-C (mmol/L)	0.99 ± 0.25	1.02 ± 0.28	1.03 ± 0.28	0.543
LDL-C (mmol/L)	2.73 ± 0.88	2.83 ± 0.91	2.41 ± 0.82 *#	0.008
eGFR (mL/min/1.73m2)	99.42 ± 18.11	100.07 ± 19.18	79.91 ± 22.78 *#	<0.001
UACR (mg/mmol)	0.65 (0.21, 1.92)	1.29 (0.39, 8.00) *	2.73 (1.02, 13.08) *	<0.001
C peptide (ng/ml)	1.39 (0.81, 2.11)	1.25 (0.81, 1.93)	1.24 (0.77, 1.66)	0.709
Hypertension	58 (46.0%)	43 (46.2%)	45 (64.3%)	0.03
CHD	8 (6.3%)	13 (14.0%)	19 (27.1%) *	<0.001
Smoking status	88 (69.8%)	62 (66.7%)	52 (74.3%)	0.103
Never	38 (30.2%)	30 (32.3%)	15 (21.4%)	
Current	0 (0.0%)	1 (1.1%)	3 (4.3%)	
Ever				
Drinking status				0.221
Never	105 (83.3%)	82 (88.2%)	63 (90.0%)	
Current	21 (16.7%)	11 (11.8%)	6 (8.6%)	
Ever	0 (0.0%)	0 (0.0%)	1 (1.4%)	
Antidiabetic agents, n (%)				
Metformin	65 (51.6%)	41 (44.1%)	32 (45.7%)	0.507
Sulfonylureas	14 (11.1%)	20 (21.5%)	17 (24.3%)	0.034
Alpha glucosidase inhibitors	17 (13.5%)	19 (20.4%)	22 (31.4%) *	0.011
SGLT2is	28 (22.2%)	25 (26.9%)	22 (31.4%)	0.359
DPP4is	21 (16.7%)	15 (16.1%)	10 (14.3%)	0.907
Thiazolidinediones	8 (6.3%)	4 (4.3%)	1 (1.4%)	0.316
Insulin	30 (23.8%)	26 (28.0%)	27 (38.6%)	0.089
GLP-1RA	17 (13.5%)	9 (9.7%)	4 (5.7%)	0.223
Antiplatelet agents	12 (9.5%)	15 (16.1%)	31 (44.3%) *#	<0.001
Nerve conduction velocity				
Left median nerve MNCV (m/s)	61.10 ± 7.14	56.92 ± 9.28 *	54.69 ± 10.72 *	<0.001
Right median nerve MNCV (m/s)	61.41 ± 9.30	56.22 ± 9.29 *	53.07 ± 13.99 *	<0.001
Left peroneal never MNCV (m/s)	49.87 ± 5.61	45.54 ± 6.49 *	44.50 ± 13.23 *	<0.001
Right peroneal never MNCV (m/s)	51.13 ± 6.10	46.22 ± 6.35 *	42.33 ± 12.38 *#	<0.001
Left median nerve SNCV (m/s)	61.66 ± 11.31	55.47 ± 13.67 *	50.90 ± 10.53 *#	<0.001
Right median nerve SNCV (m/s)	59.50 ± 10.42	53.16 ± 11.41 *	49.72 ± 15.24 *	<0.001

The data are presented as mean ± SD, numbers (%), or medians (interquartile ranges). * P<0.05 compared with patients without DPN; # P<0.05 compared with patients with sDPN.

Compared with participants without DPN, patients with DPN were significantly older, had a longer duration of diabetes, poorer glycemic control, and lower diastolic blood pressure. In addition, serum ALT, albumin, hemoglobin, eGFR, TC, and LDL-C levels were significantly lower in the DPN group. The prevalence of coronary heart disease (CHD) and hypertension was also significantly higher among patients with DPN.

As expected, both motor nerve conduction velocities (MNCVs) and sensory nerve conduction velocities (SNCVs) were significantly reduced in patients with DPN. Furthermore, patients with DPN demonstrated higher rates of alpha-glucosidase inhibitor and sulfonylurea use.

**Figure 1:**
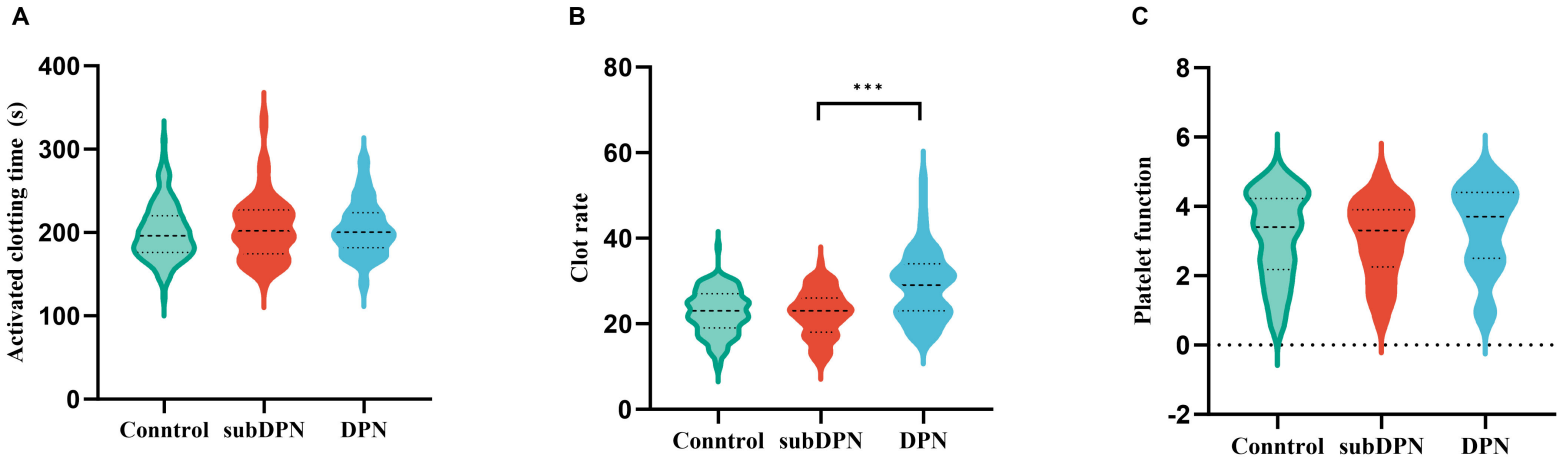
Distribution of Sonoclot parameters across different disease groups: **(A)** activated clotting time, **(B)** clot rate, and **(C)** platelet function.***p < 0.001.

Interestingly, CR levels did not differ significantly between the non-DPN and sDPN groups (**Figure 1**). However, patients with sDPN exhibited significantly lower CR levels than those with confirmed DPN, indicating that CR may help distinguish sDPN from confirmed DPN in this cross-sectional cohort.

### Correlation of CR with clinical parameters

**Figure 2:**
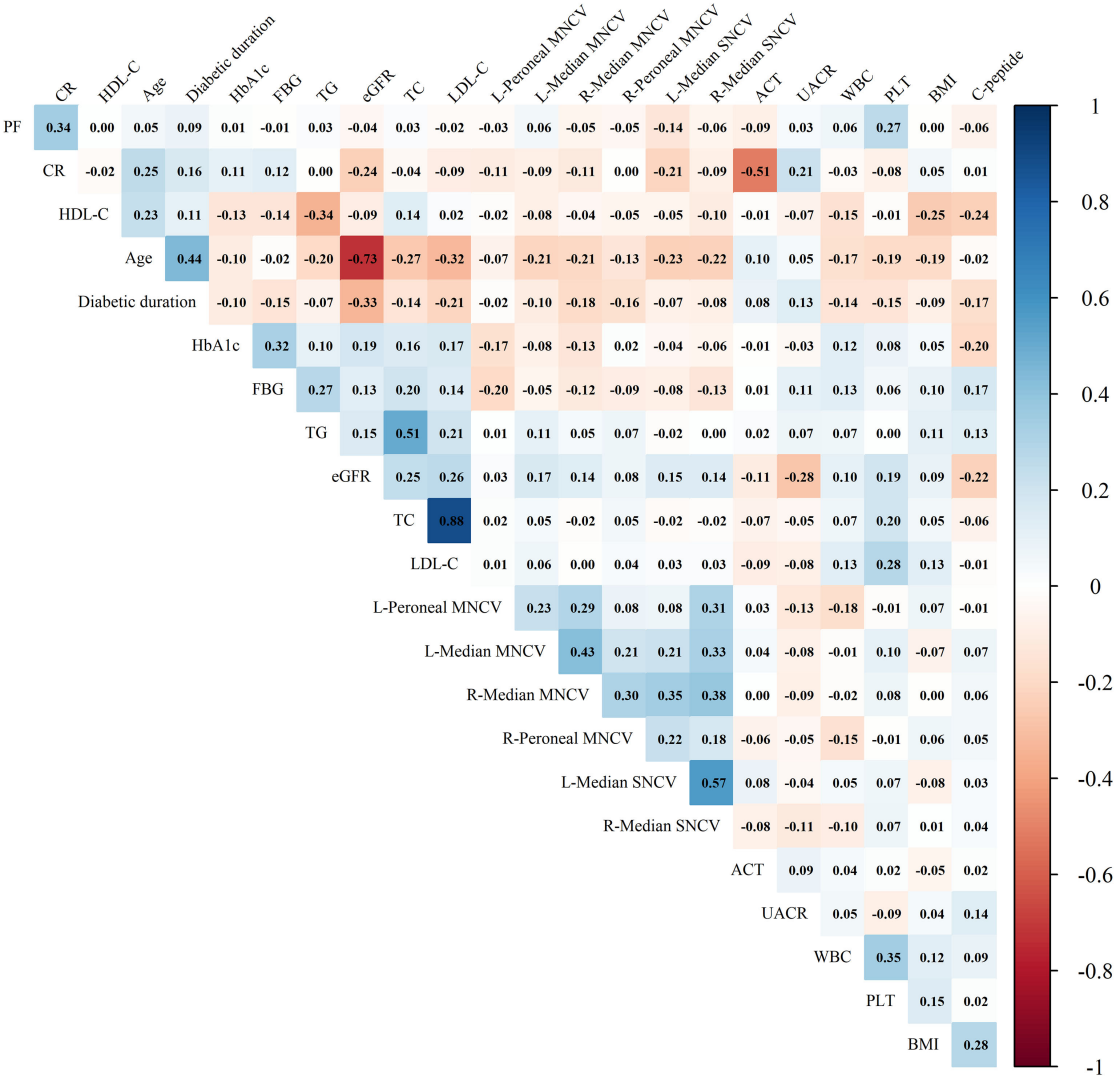
The heatmap depicts the relationship between the CR and the other variables in the entire cohort.

As shown in **Figure 2**, Spearman correlation analysis demonstrated that CR was significantly negatively correlated with left median SNCV. In contrast, CR was positively correlated with age, diabetes duration, and urinary albumin-to-creatinine ratio (UACR). Weak correlations were also observed between CR and right median MNCV as well as left peroneal MNCV, although these associations did not reach statistical significance.

### Association between CR levels and DPN

The association between CR levels and DPN was initially evaluated by treating CR as a continuous variable in multivariable logistic regression analyses (**Table 2**). Based on the directed acyclic graph framework, age, diabetes duration, HbA1c, BMI, and platelet count were identified as essential confounders requiring adjustment in analyses involving Sonoclot-derived parameters and DPN.

**Table 2:** Multivariable logistic regression analysis to explore association of CR wtih DPN.

Models	OR (95% CI)	P-value
Model I	1.168 (1.099,1.241)	<0.001
Model II	1.156 (1.078,1.240)	<0.001
Model III	1.154 (1.075,1.239)	<0.001

Model 1 was unadjusted; Model 2 was adjusted for age, diabetic duration, HbA1c; Model 3 was adjusted for age, diabetic duration, HbA1c, BMI, and platelet count.

The association between CR and DPN remained statistically significant in the fully adjusted model (Model III), which controlled for age, diabetes duration, HbA1c, BMI, and platelet count. In the fully adjusted model, higher CR was associated with higher odds of DPN, with each unit difference in CR corresponding to 15.4% higher odds of DPN (OR 1.154, 95% CI 1.075–1.239).

**Figure 3:**
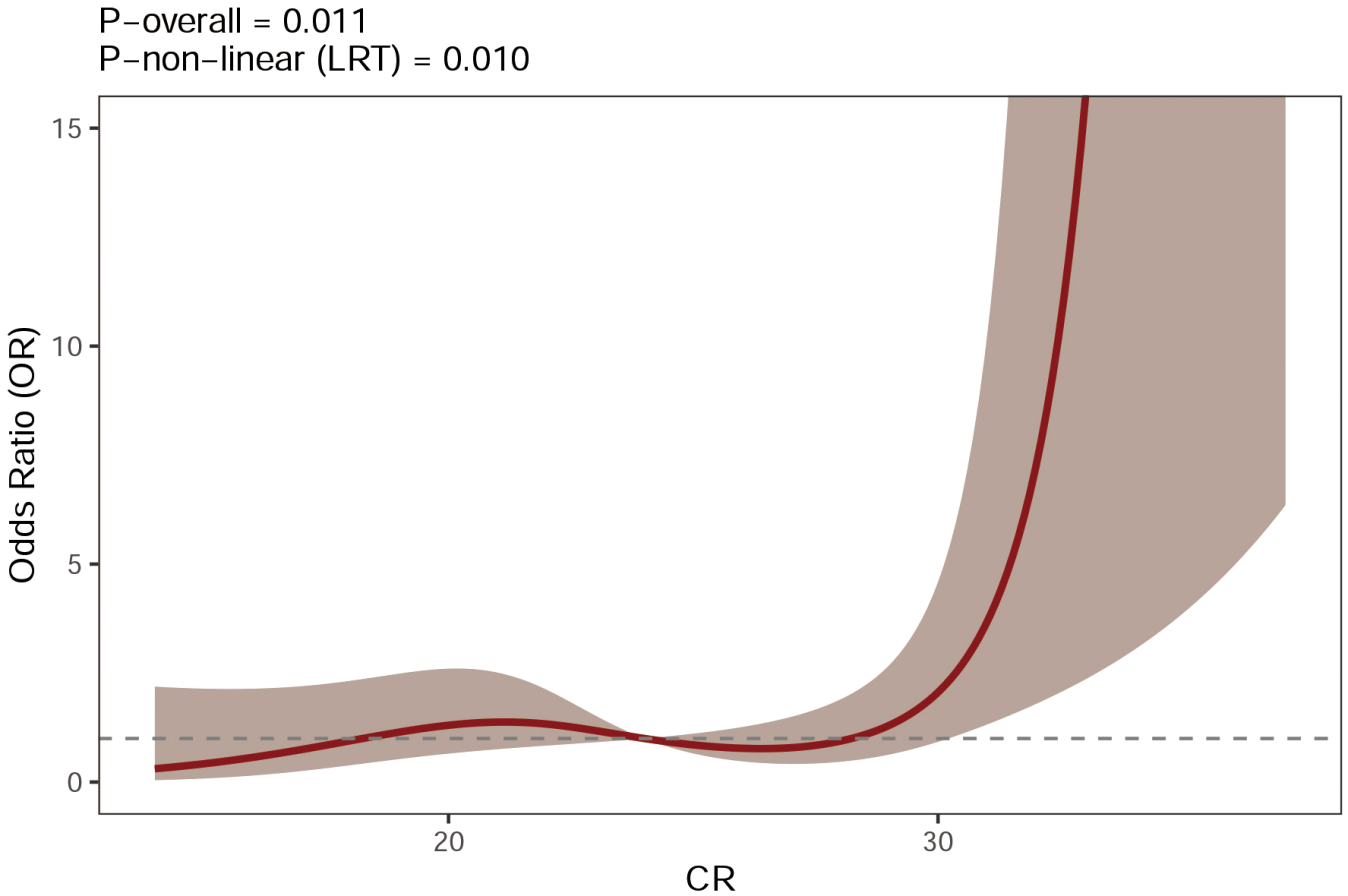
Restricted cubic spline of the nonlinear trends between the CR and DPN.

To further characterize the relationship between CR levels and the presence of DPN, restricted cubic spline regression was performed using the multivariable-adjusted model. This analysis demonstrated a significant nonlinear association between CR levels and the probability of DPN (**Figure 3**). Subsequent threshold effect analysis identified a turning point at CR = 23.983. Specifically, when CR was below 23.983, the odds of DPN showed no statistically significant change (OR: 1.191, 95% CI: 0.963–1.472, P = 0.106); however, when CR exceeded 23.983, higher CR was associated with substantially higher odds of DPN (OR: 1.448, 95% CI: 1.178–1.780, P < 0.01).

### Subgroup analysis

**Figure 4:**
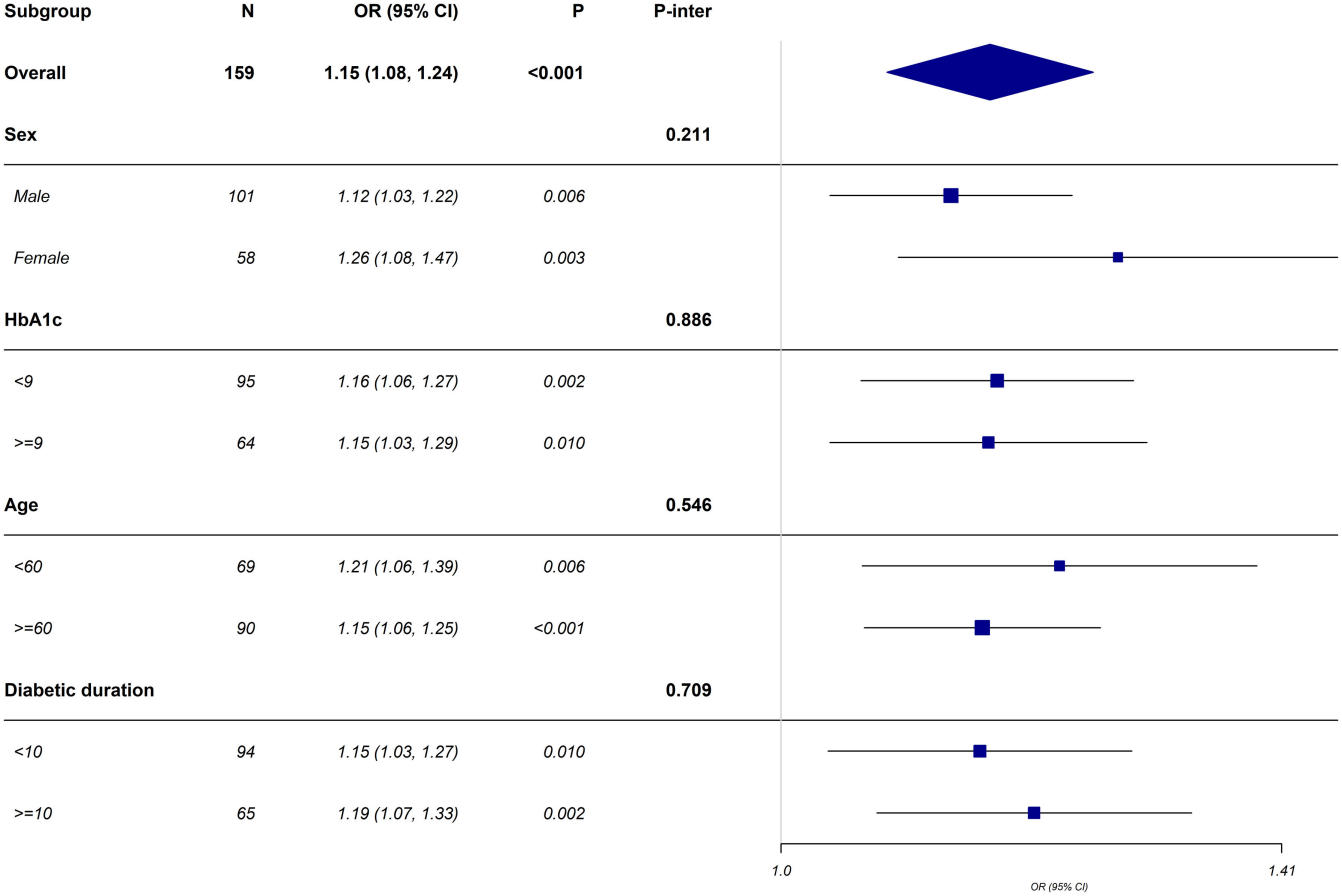
Subgroup analyses of the association between the CR and DPN.

Subgroup analyses were subsequently performed to further evaluate the association between CR and DPN across clinically relevant populations (**Figure 4**). All models were adjusted for age, diabetes duration, HbA1c, BMI, and platelet count.

The positive association between CR levels and the presence of DPN remained consistent across most subgroups, including those stratified by sex, age (>60 years), diabetes duration (>5 years), HbA1c level (≤9%), and the presence or absence of hypertension or CHD. No statistically significant interactions were observed, supporting the consistency of the observed association across these subgroups.

### Diagnostic performance of CR for DPN

**Figure 5:**
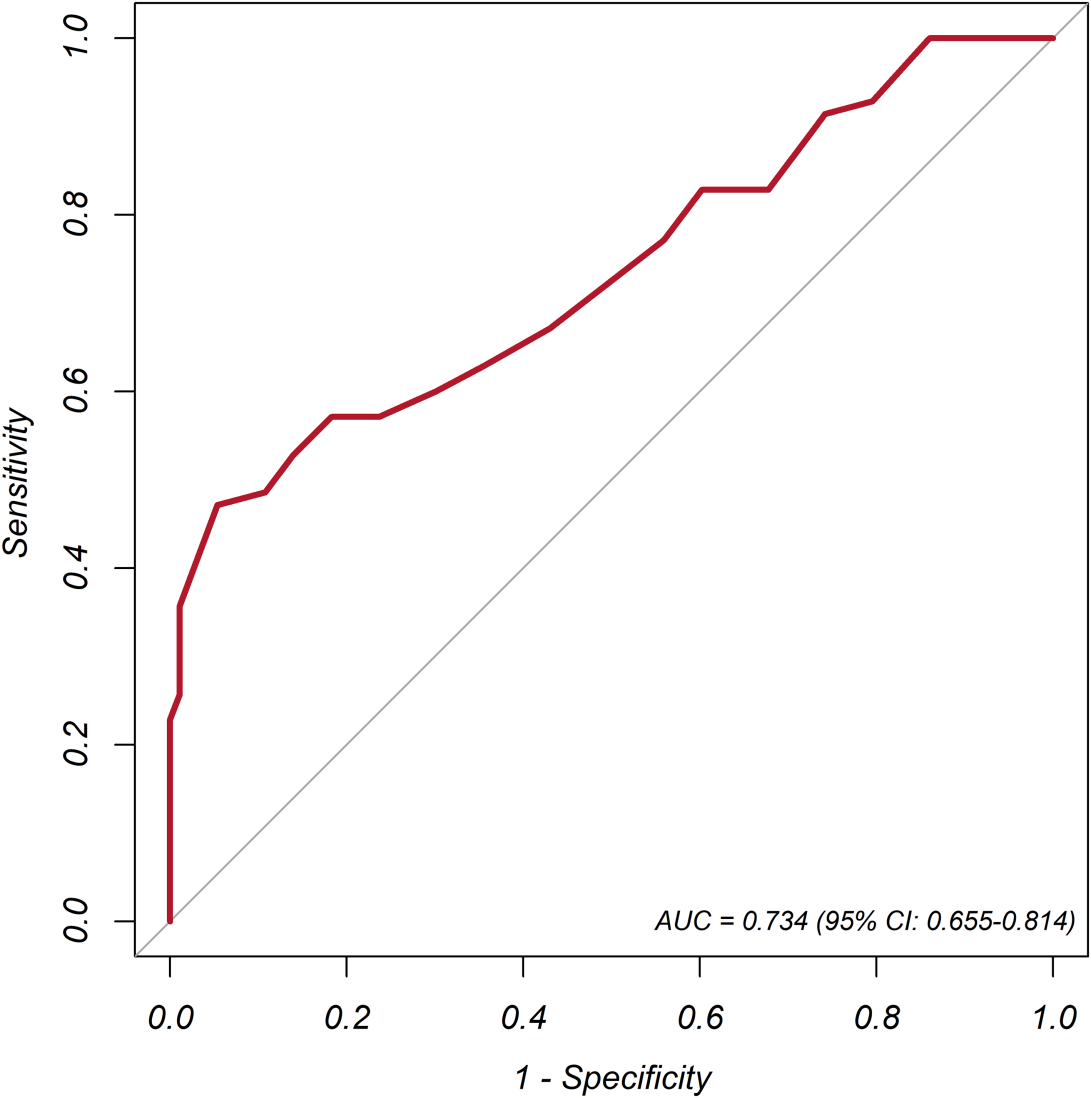
The discriminatory performance of CR.

ROC curve analysis was performed to assess the diagnostic performance of CR for DPN (**Figure 5**). The area under the curve (AUC) for CR was 0.729. The optimal cutoff value for CR was 31.1, corresponding to a modest sensitivity of 47.1% and a specificity of 98.9%.

## Discussion

Coagulation dysfunction plays a pivotal role in the development of diabetic microvascular complications. T2DM is characterized by a chronic prothrombotic state accompanied by systemic hypercoagulability and persistent low-grade inflammation, all of which contribute to endothelial injury, impaired microvascular perfusion, and progressive neurovascular damage (18–20).

Our findings demonstrated distinct differences in Sonoclot-derived clot rate (CR) across DPN status. Specifically, CR levels remained comparable between patients without DPN and those with subclinical DPN, whereas a marked elevation in CR was observed in patients with confirmed DPN. After adjustment for major confounding variables, CR remained independently associated with DPN, with each unit difference corresponding to 15.4% higher odds of DPN (OR 1.154, 95% CI 1.075–1.239). Furthermore, restricted cubic spline analysis demonstrated a significant nonlinear association between CR levels and the probability of DPN. ROC analysis identified an optimal CR cutoff value of 31.1, which yielded excellent specificity (98.9%) despite modest sensitivity (47.1%).

These findings provide important insight into the cross-sectional relationship between hypercoagulability and diabetic neuropathy status. Previous studies have consistently demonstrated that coagulation abnormalities, particularly elevated fibrinogen and D-dimer levels, are associated with both the development and severity of DPN (9). However, conventional plasma-based coagulation assays, including PT and APTT, incompletely reflect the complex interactions among platelets, fibrin formation, and coagulation factors occurring *in vivo*, thereby limiting their ability to characterize whole-blood thrombotic activity (21). To overcome these limitations, recent investigations have increasingly focused on viscoelastic testing modalities such as TEG (22). Zhuang et al. reported that TEG-derived parameters reflecting fibrinogen activity, including angle α and k-value, were significantly altered during the early subclinical stage of DPN (12, 13). In contrast, the present study demonstrated that Sonoclot-derived CR remained relatively stable in patients with sDPN but was substantially higher in those with confirmed DPN. This discrepancy may reflect differences in study design, disease stage, and measurement principles. The study by Zhuang et al. was longitudinal and evaluated baseline TEG-derived indices as predictors of incident DPN, whereas our study was cross-sectional and assessed Sonoclot-derived parameters according to prevalent DPN status. In addition, TEG primarily reflects macroscopic clot formation and fibrin cross-linking dynamics, which are strongly influenced by overall fibrinogen availability (23, 24). Conversely, the Sonoclot system utilizes a vertically oscillating probe that is particularly sensitive to early alterations in blood viscosity, fibrin polymerization kinetics, and platelet–fibrin interactions occurring during the initial stages of clot formation (25). Therefore, TEG and Sonoclot should be considered complementary rather than interchangeable approaches for evaluating coagulation abnormalities in DPN.

The absence of elevated CR in sDPN despite previously reported early TEG abnormalities suggests that generalized hypercoagulability may emerge relatively early during diabetic neuropathy progression, whereas the accelerated fibrin polymerization detected by Sonoclot CR may predominantly reflect the transition from compensated microvascular dysfunction to irreversible ischemic nerve injury. During the subclinical stage, endothelial dysfunction may initially be partially counterbalanced by endogenous fibrinolytic activity and compensatory microvascular recruitment. However, persistent glycemic toxicity and accumulation of advanced glycation end-products may be associated with alterations in fibrinolytic degradation (26). Simultaneously, endothelial injury may enhance tissue factor exposure and localized thrombosis, ultimately leading to microvascular occlusion, nerve ischemia, and clinically overt neuropathy.

Accordingly, the present study extends previous observations by suggesting that Sonoclot-derived CR may represent a highly specific biomarker associated with clinically confirmed DPN rather than sDPN. Unlike conventional coagulation markers or earlier viscoelastic indices that broadly reflect systemic hypercoagulability, elevated CR may help identify patients with clinically significant DPN characterized by more prominent endoneurial ischemic injury. The exceptionally high specificity observed in the present study further supports the potential utility of CR as a confirmatory biomarker for clinically established DPN. However, the modest sensitivity observed in our study indicates that CR alone would miss a substantial proportion of DPN cases and therefore should not be considered an independent screening tool. It is crucial to note that patients with confirmed DPN had a higher prevalence of CHD and antiplatelet agent use. Although Sonoclot CR predominantly measures fibrin polymerization, concurrent antithrombotic therapies and vascular comorbidities such as CHD and nephropathy may influence whole-blood viscoelasticity, endothelial dysfunction and prothrombotic activation representing a potential source of residual confounding (27–29).

Several limitations should nevertheless be acknowledged. First, the cross-sectional design precludes causal or temporal inference. Residual confounding and the absence of longitudinal follow-up further limit assessment of the predictive value of CR for incident DPN. Second, this single-center study was conducted in a tertiary-care inpatient population, which may introduce selection bias and limit generalizability. Moreover, the ROC-derived CR cutoff lacks external validation. Therefore, this threshold should be considered exploratory. Future studies should therefore explore integrated diagnostic strategies combining Sonoclot-derived hemostatic parameters with highly sensitive structural assessments (30). Third, Sonoclot and TEG were not directly compared in the same cohort. Future prospective multicenter studies incorporating external validation and head-to-head comparisons are warranted.

In conclusion, our cross-sectional findings demonstrate that an elevated Sonoclot-derived CR is independently associated with clinically established DPN. With its high specificity, CR may serve as a potential confirmatory marker for overt DPN in patients with T2DM. Prospective, longitudinal cohort studies with external validation are strongly recommended to clarify temporality, evaluate potential causal relationships, and confirm clinical utility.

## Data Availability

The raw data supporting the conclusions of this article will be made available by the authors, without undue reservation.
